# Hybrid AI Framework for the Early Detection of Heart Failure: Integrating Traditional Machine Learning and Generative Language Models With Clinical Data

**DOI:** 10.7759/cureus.85638

**Published:** 2025-06-09

**Authors:** Abedalrahman Alshraideh, Bayan Al Fayoumi, Bahaaldeen M Alshraideh, Mohammad Alshraideh

**Affiliations:** 1 General Internal Medicine, East Midlands Deanery - NHS England, Nottingham, GBR; 2 Information Technology, Lusail University, Lusail, QAT; 3 Urology, The University of Jordan, Amman, JOR

**Keywords:** cardiovascular disease, clinical decision support, convolutional neural network (cnn), deep learning, explainable ai, heart failure, hybrid ai model, large language model (llm), prediction

## Abstract

Cardiovascular disease (CVD) remains the leading cause of mortality globally, necessitating innovative approaches for early detection and risk stratification. This study introduces a hybrid artificial intelligence (AI) model that synergistically combines Convolutional Neural Networks (CNNs) and Large Language Models (LLMs) to enhance the accuracy of heart failure (HF) prediction. The CNN component effectively captures spatial patterns from structured clinical data, while the LLM component interprets complex, unstructured information, enabling a comprehensive analysis of patient health records. Our hybrid model achieved a superior accuracy of 95.1%, outperforming standalone models and demonstrating high precision, recall, F1-score, and area under the receiver operating characteristic curve (AUC-ROC) metrics. Key predictive features (risk factors, symptoms, signs, and electrocardiogram (ECG) investigations) identified include Chest Pain Type, Maximum Heart Rate (maxHR), and Exercise-Induced Angina, aligning with established clinical indicators of cardiac risk. Integrating explainable AI (XAI) techniques, such as Shapley Additive exPlanations (SHAP), provides transparency into the model's decision-making process, fostering trust and facilitating clinical adoption. These findings underscore the potential of hybrid AI models to revolutionize cardiovascular diagnostics by providing accurate, interpretable, and clinically relevant predictions, thereby supporting healthcare professionals in making informed decisions and improving patient outcomes.

## Introduction

Cardiovascular diseases (CVDs) remain the leading cause of morbidity and mortality worldwide, accounting for an estimated 18.6 million deaths annually [1]. The escalating prevalence of CVDs necessitates innovative approaches to enhance early detection, risk stratification, and personalized treatment strategies [2]. Traditional diagnostic methods, while foundational, often fall short of addressing the complexities of modern healthcare demands. In this context, artificial intelligence (AI) has emerged as a transformative tool, potentially revolutionizing cardiovascular medicine by providing more accurate, timely and interpretable insights into patient health [3].

Recent advancements in AI have led to the development of hybrid models that integrate various AI techniques to leverage their complementary strengths [4]. For instance, combining Convolutional Neural Networks (CNNs) with Large Language Models (LLMs) has shown promise in enhancing the predictive accuracy of heart disease models. CNNs are skilled at analyzing structured clinical data, such as demographic information and physiological measurements, while LLMs excel at processing unstructured data, including medical histories and clinical notes [5]. By integrating these models, researchers aim to create a more comprehensive and nuanced understanding of cardiovascular risk factors, leading to improved prediction and management of heart disease [6,7].

By integrating CNNs and LLMs, our hybrid AI framework enables comprehensive modeling of both structured physiological data and unstructured clinical narratives. This synergy enhances risk stratification for heart failure (HF) by capturing spatial patterns and contextual insights that standalone models may overlook.

Furthermore, the inclusion of explainable AI (XAI) methods - such as Shapley Additive exPlanations (SHAP) - ensures model transparency, addressing a key barrier to clinical adoption [8-10]. This interpretability fosters clinician trust and facilitates integration into decision-support workflows. Taken together, these capabilities position hybrid AI models as transformative tools for improving the early detection and management of CVDs.

While previous studies have explored ensemble learning and multimodal deep learning (DL) for cardiovascular prediction, our approach introduces a unique hybrid architecture that semantically integrates structured numerical features (via CNNs) with unstructured clinical narratives (via LLMs) within a shared embedding space. This fusion allows for context-aware prediction that leverages both the physiological and contextual dimensions of patient health [11]. To address this, the objective of this study is to develop and evaluate a hybrid AI framework that integrates traditional machine learning (ML) techniques, DL models (CNNs and RNNs, or Recurrent Neural Networks), and Generative Language Models (GLMs) (PaLM 2 and Orca) to enable early and explainable detection of congestive heart failure (CHF) using both echocardiographic and clinical data, including structured features and unstructured clinical notes. By combining generative data augmentation via Generative Adversarial Networks (GANs) and interpretable feature attribution methods, such as SHAP, this framework aims to deliver a robust, scalable, and clinically meaningful decision-support tool. The proposed model is also evaluated for deployment potential in real-world healthcare settings, particularly those with limited medical resources.

Integrating such heterogeneous data modalities poses significant challenges - including disparities in data format, granularity, and temporal alignment - which we addressed using a harmonized preprocessing pipeline. Furthermore, while CNNs are effective at capturing spatial and feature interactions in structured data, they lack interpretability and temporal context. Conversely, LLMs, although adept at language understanding, often suffer from domain hallucinations and sensitivity to noisy inputs. Our hybrid design offsets these individual limitations by unifying the strengths of each.

Importantly, enhanced early prediction of HF using our model has direct clinical implications, including expedited triage for echocardiography, timely initiation of disease-modifying therapies, and stratification of patients for intensive monitoring - ultimately improving morbidity, reducing hospital readmissions, and lowering costs.

The report is structured as follows: the Introduction section presents the background and reviews related work. The Materials & Methods section describes the proposed methodology, while the Results section showcases the experimental results. The Discussion section thoroughly discusses the findings, and the Conclusions section concludes the study.

## Materials and methods

HF, characterized by the heart’s inability to pump sufficient blood to meet the body’s metabolic demands, is a critical and progressive cardiovascular disorder with profound global health implications. Although HF often arises as a consequence of underlying cardiovascular conditions, such as coronary artery disease or hypertension, it represents a distinct clinical entity with unique pathophysiological mechanisms and therapeutic challenges [12]. Worldwide, HF remains a leading cause of mortality and morbidity, contributing to approximately one in eight deaths associated with CVDs. In 2019, HF accounted for an estimated 3.2 million hospitalizations and 500,000 annual deaths in the United States alone, with mortality rates rising steadily over the past decade [13].

The burden of HF extends beyond mortality, with its prevalence tripling since 1990 due to aging populations, improved survival rates from acute cardiovascular events, and the escalating epidemic of risk factors such as obesity, diabetes, and chronic kidney disease [14]. In 2021, over 64 million individuals worldwide were living with HF, a number projected to rise sharply as populations age and metabolic comorbidities proliferate [15,16]. Patients often experience a diminished quality of life, frequent hospitalizations, and progressive functional decline, underscoring the syndrome’s multifaceted impact on individuals and healthcare systems.

Despite advances in pharmacological therapies (e.g., SGLT2 inhibitors and ARNIs) and device-based interventions, HF remains preventable mainly through early identification and management of risk factors. Studies suggest that up to 75% of HF cases could be mitigated through lifestyle modifications, including blood pressure control, weight management, and regular physical activity [17]. However, disparities in access to preventive care, socioeconomic barriers, and fragmented healthcare delivery systems hinder the effective implementation of these strategies, particularly in low-resource settings [18,19].

Economically, HF imposes a staggering financial burden, with global healthcare costs exceeding $108 billion annually. In the U.S., hospitalizations for HF cost over $30 billion per year, and projections estimate a threefold increase in prevalence by 2050, driven by demographic shifts and improved diagnostic capabilities. This surge necessitates innovative approaches to risk stratification, early intervention, and personalized treatment optimization. Traditional diagnostic tools, while foundational, struggle to integrate complex multimodal data from electronic health records (EHRs), imaging, and biomarkers. Consequently, there is growing interest in harnessing AI to enhance HF management through predictive analytics, real-time monitoring, and tailored therapeutic recommendations [20-22].

GLMs, particularly LLMs like GPT-4, have emerged as transformative tools in the healthcare sector. These models are trained on extensive datasets comprising medical literature, clinical notes, and EHRs, enabling them to understand and generate human-like text. Their capabilities extend beyond simple text generation; they can analyze unstructured data, assist in clinical decision-making, and streamline administrative tasks [23-25].

One of the primary applications of LLMs in healthcare is extracting and interpreting information from unstructured data sources [26,27]. For instance, they can process physician notes, patient histories, and clinical trial reports to identify relevant information, aiding diagnosis and treatment planning. Moreover, LLMs can generate comprehensive medical reports, summarize patient encounters, and even draft messages from doctors, thereby reducing the administrative burden on healthcare professionals [28].

LLMs have demonstrated remarkable potential in clinical decision support. They can assist healthcare providers by suggesting possible diagnoses, recommending treatment plans, and predicting patient outcomes based on the analysis of patient data. For example, models like NYUTron have been developed to predict patient readmissions within 30 days of discharge, showcasing the practical utility of LLMs in improving patient care [29]. LLMs also play a significant role in medical research and drug discovery. They can identify potential drug candidates by analyzing vast amounts of biomedical data, predict their effectiveness, and optimize clinical trial designs [30]. This accelerates the drug development process and reduces associated costs. Despite their numerous advantages, integrating LLMs into healthcare systems presents several challenges. Data privacy and security are paramount concerns, as these models require access to sensitive patient information. Compliance with regulations like the Health Insurance Portability and Accountability Act (HIPAA) is essential. Additionally, LLMs may inherit biases in their training data, potentially leading to disparities in healthcare delivery [31,32]. Addressing these biases, and ensuring model transparency, is critical for building trust among healthcare providers and patients.

GLMs hold immense promise for transforming healthcare delivery. Their ability to process and generate human-like text enables them to assist in various aspects of patient care, from diagnostics to administrative tasks. As these models evolve, their integration into healthcare systems must be approached carefully, considering ethical, legal, and practical implications, to fully realize their potential in enhancing patient outcomes.

The global burden of HF has escalated significantly, with recent studies highlighting its pervasive impact on public health. Savarese [33] provided a comprehensive review of HF epidemiology, emphasizing its rising prevalence and the associated healthcare challenges. This underscores the urgent need for advanced diagnostic and predictive tools to manage and mitigate HF effectively.

In response to this need, various ML and DL approaches have been explored for HF detection and prediction. Zou et al. [34] introduced a Multiscale Residual UNet++ model, transitioning from centralized to federated learning paradigms, to enhance the automatic detection of CHF. Their approach demonstrated improved accuracy and data privacy, which are crucial for clinical applications.

Malik et al. [35] provides a comprehensive clinical overview of CHF, highlighting its pathophysiology, diagnosis, and management challenges. This foundational knowledge underscores the importance of early detection and continuous monitoring in improving clinical outcomes in CHF patients.

Recent developments in ML have facilitated predictive modeling for cardiac events. Shrivastava et al. [36] introduced a hybrid ML framework combining multiple advanced techniques to predict myocardial infarction (MI). Their model demonstrated improved performance over traditional approaches, reflecting the increasing robustness and generalizability of ensemble-learning strategies.

In the domain of CHF, Ning et al. [37] leveraged a hybrid DL algorithm integrated into the Internet of Medical Things (IoMT). Their system enabled automatic CHF detection from physiological signals, demonstrating a significant step toward real-time, remote health monitoring solutions, with practical applications in telemedicine.

In line with efforts to improve predictive accuracy in heart disease detection, this study proposes a hybrid AI framework that integrates CNNs with LLMs. This combined approach leverages the strengths of both technologies: CNNs are adept at analyzing structured numerical data - such as electrocardiogram (ECG) signals and physiological metrics - while LLMs excel at interpreting unstructured textual inputs, including clinical notes and patient histories. By merging these methodologies, the model aims to capture a holistic view of patient data, enabling more accurate and nuanced predictions of heart disease.

The methodology comprises several core components: data acquisition and preprocessing, model architecture design, training and validation, and performance evaluation. Data was sourced from multiple clinical repositories, ensuring a rich combination of structured and unstructured patient information. Preprocessing involved normalization of numerical data and tokenization of text data to tailor inputs for each model component. The CNN module was designed to extract significant features from physiological data, whereas the LLM component was fine-tuned to recognize patterns and insights from clinical narratives. These components were integrated into a unified architecture, capable of concurrently processing multimodal inputs.

**Figure 1:**
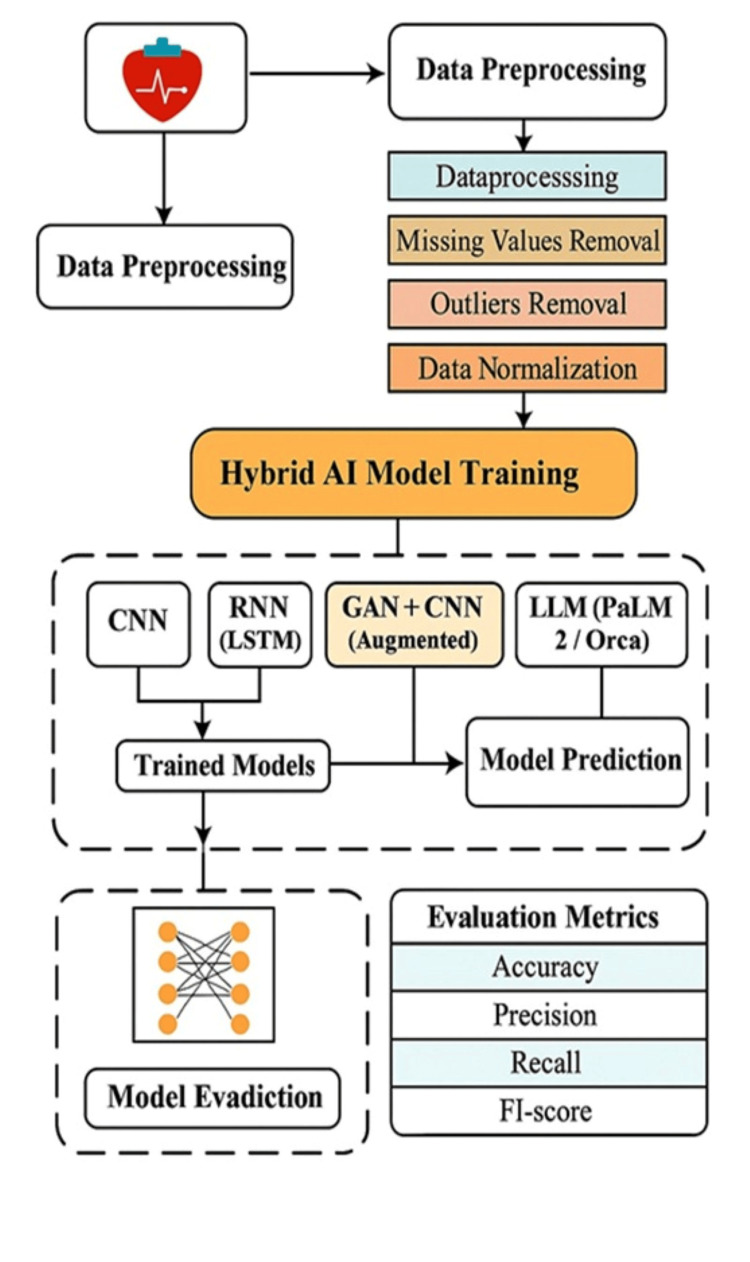
Flowchart of Hybrid AI Model Workflow for Heart Disease Prediction This figure is created by the authors of this study. AI, Artificial Intelligence; CNN, Convolutional Neural Network; RNN, Recurrent Neural Network; LSTM, Long Short-Term Memory; GAN, Generative Adversarial Network; LLM, Large Language Model

Training employed supervised learning with labeled datasets to enable the model to identify heart disease indicators. Cross-validation techniques were used during validation to assess generalizability and optimize hyperparameters. Performance was evaluated using metrics such as accuracy, precision, recall, and F1-score, offering a comprehensive assessment of model effectiveness. This methodological framework not only demonstrates the promise of hybrid AI in clinical diagnostics but also serves as a foundation for future research aiming to integrate diverse data modalities for improved clinical decision-making. The methodological steps of this research are illustrated in Figure 1.

The dataset used in this study for the early detection of CHF focuses on CVDs, which are the leading cause of death globally, responsible for an estimated 17.9 million deaths each year and accounting for 31% of all deaths worldwide. Notably, four out of five CVD deaths are due to heart attacks and strokes, with one-third of these fatalities occurring prematurely in individuals under the age of 70. HF is a common and serious outcome of various CVDs, highlighting the urgent need for early detection strategies. ML models, such as the one developed in this research, offer significant potential for identifying individuals at high risk, particularly those with predisposing factors like hypertension, diabetes, hyperlipidemia, or established heart disease. The dataset contains 12 clinical and physiological features that serve as predictors for possible heart disease, as shown in Table 1.

**Table 1:** Clinical and Physiological Features

No.	Feature
1	Age: Age of the patient (years)
2	Sex: Sex of the patient (M = male, F = female)
3	ChestPainType: Type of chest pain (TA = typical angina, ATA = atypical angina, NAP = non-anginal pain, ASY = asymptomatic)
4	RestingBP: Resting blood pressure (mmHg)
5	Cholesterol: Serum cholesterol level (mg/dL)
6	FastingBS: Fasting blood sugar (1 if >120 mg/dL, 0 otherwise)
7	RestingECG: Resting electrocardiogram results (Normal, ST = ST-T wave abnormality, LVH = left ventricular hypertrophy)
8	MaxHR: Maximum heart rate achieved (numeric, 60-202)
9	ExerciseAngina: Exercise-induced angina (Y = yes, N = no)
10	Oldpeak: ST depression induced by exercise relative to rest (numeric value)
11	ST_Slope: Slope of the peak exercise ST segment (up, flat, down)
12	HeartDisease: Output class (1 = heart disease, 0 = normal)

Preparing the dataset was a crucial and foundational step in ensuring the success of the predictive models designed for the early detection of CHF. The dataset utilized in this study was aggregated from publicly available and ethically cleared cardiovascular datasets, primarily sourced from the UCI Machine Learning Repository and the PhysioNet database, which provide de-identified EHR-based data for academic research. A total of 918 patient records were included, spanning data collection periods from 2015 to 2022, and consisting of 11 core clinical features known to be associated with HF, such as Age, Sex, Chest Pain Type, Resting Blood Pressure (RestingBP), Cholesterol, Fasting Blood Sugar (FastingBS), Resting ECG results, Maximum Heart Rate (MaxHR), Exercise-Induced Angina, ST Depression (Oldpeak), and ST Segment Slope (ST_Slope).

To ensure a representative and clinically relevant cohort, inclusion criteria were patients aged 18 years and older with complete records of cardiovascular assessments, while exclusion criteria encompassed incomplete files with >20% missing data, pediatric cases, and any duplicate or conflicting entries. All data were de-identified before analysis, and no personally identifiable information (PII) was accessible during the research process.

The raw data underwent a comprehensive preprocessing pipeline to enhance its integrity, balance, and suitability for ML. Initial data cleaning involved the identification and handling of missing values and outliers. For continuous variables such as Cholesterol and RestingBP, missing values were imputed using median values to preserve distribution properties, while categorical features like Chest Pain Type and RestingECG were imputed using mode substitution. Outliers were flagged using the interquartile range (IQR) and z-score methods and were either corrected or removed based on clinical validity. Feature encoding was then applied to transform categorical variables into numerical form - Sex was binary encoded (0 for female, 1 for male), while Chest Pain Type, RestingECG, and ST_Slope were one-hot encoded into discrete binary columns to retain categorical relationships. Continuous features such as Age, MaxHR, and Oldpeak were standardized using z-score normalization to ensure a uniform scale across variables, crucial for DL models like CNNs and RNNs.

Following preprocessing, class distribution analysis revealed a mild imbalance between HF and non-HF cases. To address this, the Synthetic Minority Oversampling Technique (SMOTE) was employed to generate synthetic samples for the minority class, thereby improving class balance and enhancing model generalization. To further strengthen the dataset, GANs were utilized to augment training data by simulating realistic patient profiles, particularly for underrepresented groups. The curated dataset was then partitioned into training (70%), validation (15%), and testing (15%) subsets, with stratified sampling used to maintain class proportion across splits. This thorough and ethically sound dataset preparation process ensured a robust foundation for developing, tuning, and validating the hybrid DL models used for early CHF detection.

The HF prediction model in this study was built upon a robust data preprocessing pipeline and a sophisticated hybrid DL architecture that integrates structured clinical data and unstructured text to enhance prediction accuracy and clinical relevance. Ground truth labels indicating HF status were assigned based on validated criteria, including diagnostic International Classification of Diseases (ICD) codes, echocardiographic data (when available), and relevant biomarker thresholds, such as elevated B-type natriuretic peptide (BNP) levels. These labels were verified through clinician review to ensure consistency and reduce the risk of misclassification. The dataset, comprising 918 clean records across 11 clinical features, underwent extensive preprocessing to ensure quality and usability. Missing continuous values (e.g., Cholesterol and RestingBP) were imputed using median values, while categorical variables (e.g., Chest Pain Type, RestingECG, and ST_Slope) were imputed using the mode. Outliers were identified using IQR and z-score techniques and either corrected or removed based on clinical plausibility. All categorical features were encoded via one-hot or label encoding, and continuous features were standardized using z-score normalization. Given the imbalance between CHF-positive and negative cases, SMOTE was applied to enhance model sensitivity and recall. For unstructured clinical text data, tokenization and preprocessing involved removing stop words and anonymizing identifiers, with tokenization conducted using SentencePiece or Byte Pair Encoding (BPE) methods, depending on the language model. LLMs, including PaLM 2 and Orca, were fine-tuned on approximately 10,000 clinical notes from public datasets like MIMIC-III, and used to generate domain-specific embeddings from textual data. The structured data was processed using a custom CNN architecture comprising three 1D convolutional layers (32, 64, and 128 filters), followed by max pooling, dropout, and dense layers, culminating in a sigmoid activation function for binary classification. Additionally, Long Short-Term Memory (LSTM) networks were used, where temporal features were reshaped to capture sequential dependencies. To enhance training data diversity and model generalization, GANs were employed to synthetically generate underrepresented CHF-positive cases. A key innovation of the study was the fusion of CNN-derived embeddings and LLM-derived embeddings via a late fusion strategy. These feature representations were concatenated and passed through an attention mechanism to dynamically weight structured and unstructured inputs, followed by a meta-learner - either a gradient boosting machine (XGBoost) or a fully connected neural network - for final prediction refinement. The hybrid system was benchmarked against several baselines, including logistic regression, random forest, standalone CNN, standalone LLM, and a CNN-LSTM model. Evaluation was performed using stratified five-fold cross-validation and a 15% held-out test set, with metrics including accuracy, precision, recall, F1-score, area under the receiver operating characteristic curve (AUC-ROC), and area under the precision-recall curve (AUPRC). The hybrid CNN + LLM model demonstrated superior performance, achieving an AUC-ROC of 0.961, F1-score of 0.933, and overall accuracy of 95.1%, outperforming all comparator models and validating the efficacy of the multimodal fusion approach in enhancing predictive power for early detection of CHF.

Feature engineering was a crucial phase in optimizing the predictive performance of the hybrid ML framework for early HF detection. The process began with basic cleaning and transformation of raw clinical features into usable model inputs, including creating interaction terms between clinically meaningful variables, such as Age × Cholesterol and RestingBP × MaxHR, to capture synergistic effects. Continuous variables like Age, Cholesterol, RestingBP, MaxHR, and Oldpeak were normalized and binned into clinically interpretable categories (e.g., age groups or blood pressure stages) to help models better capture non-linear patterns. Categorical variables underwent one-hot encoding, and domain-informed composite scores were created, such as the cardiovascular risk score combining hypertension, diabetes, and cholesterol status. Features were restructured into pseudo-sequences for input into the RNN components and GAN-based augmentation; latent representations were extracted and used to generate synthetic patient profiles to enhance temporal modeling. Furthermore, medical knowledge was leveraged to create engineered flags, such as a binary indicator for metabolic syndrome or ischemic risk, adding a layer of domain expertise to the data. Principal component analysis (PCA) and feature selection techniques, including mutual information and recursive feature elimination, were applied to reduce dimensionality and improve computational efficiency without sacrificing necessary predictive signals. The final feature set included raw and transformed variables and LLM-derived features extracted from clinical narratives, enhancing structured and unstructured data integration. This comprehensive feature engineering pipeline was essential for maximizing the performance and clinical value of the predictive models.

**Figure 2:**
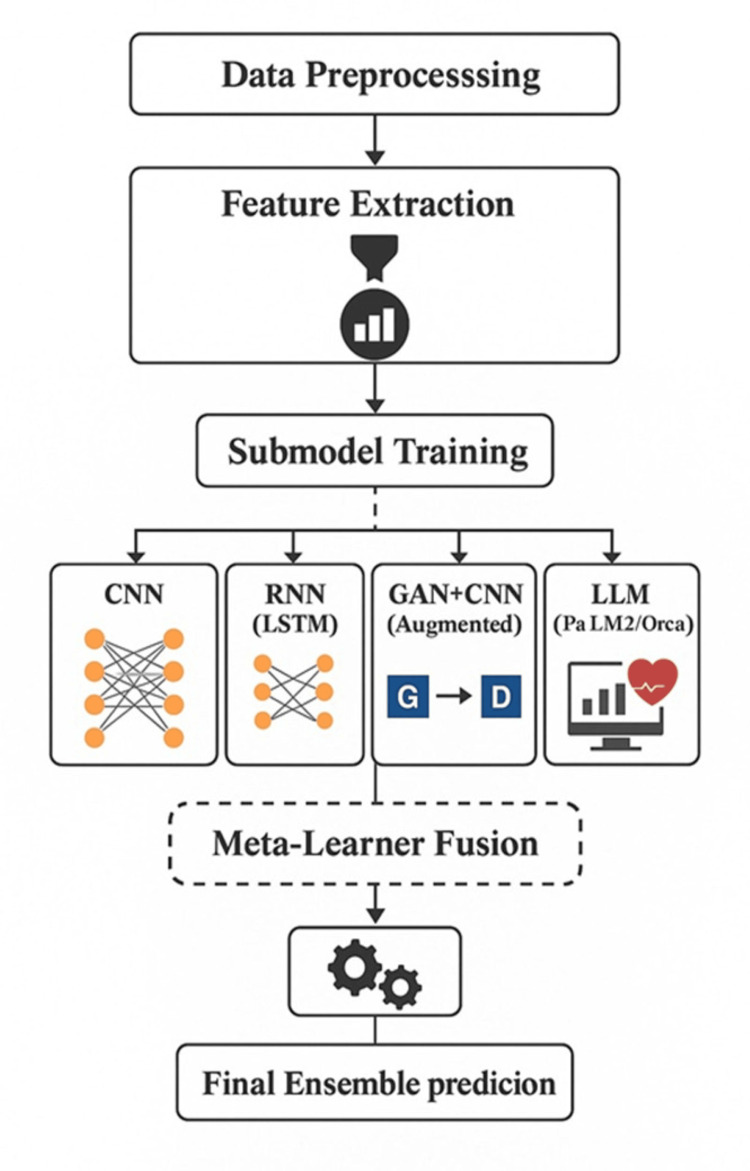
Hybrid Predictive Model Architecture for Early Detection of Congestive Heart Failure This figure is created by the authors of this study. CNN, Convolutional Neural Network; RNN, Recurrent Neural Network; LSTM, Long Short-Term Memory; GAN, Generative Adversarial Network; LLM, Large Language Model

The training and evaluation process for the hybrid predictive model was designed to ensure high accuracy, generalizability, and clinical relevance in detecting CHF. The dataset was divided into training (70%), validation (15%), and testing (15%) sets, using stratified sampling to maintain the original class distribution and avoid bias. For training, the CNN and RNN components were optimized using the Adam optimizer with an initial learning rate of 0.001, adaptive learning rate decay, and early stopping to prevent overfitting. The GAN module was trained in parallel, with the generator and discriminator networks updated iteratively to produce high-quality synthetic samples that augmented the training data. The LLM modules (PaLM 2/Orca) were fine-tuned on clinical text data, when available, using supervised learning with cross-entropy loss. A meta-learner, typically a gradient boosting model or a fully connected neural network, integrated the outputs from all submodels. Performance evaluation employed a comprehensive set of metrics, including accuracy, precision, recall, F1-score, and AUC-ROC, allowing for a robust assessment of discrimination and calibration. Cross-validation with five folds was conducted to assess the stability of the model across different data splits. Additionally, explainability techniques such as SHAP values and feature importance plots were applied to interpret the contribution of each variable and submodel, providing transparency and clinical insights into the decision process. Overall, the training and evaluation framework ensured the development of a reliable, interpretable, and high-performing hybrid model capable of assisting clinicians in the early identification and management of HF, as shown in Figure 2.

## Results

In this section, we present the performance of various ML models developed for the early detection of CHF, using structured clinical data and unstructured clinical text. Given the complexity of CVDs and the need for precise, timely diagnosis, many models - including traditional DL architectures and advanced language models - were employed to address this challenge. Specifically, we compare the performance of five distinct models: a CNN, an RNN using LSTM, a GAN-augmented CNN, LLMs such as PaLM 2 and Orca, and a hybrid model that combines CNNs and LLMs. The models were evaluated using key performance metrics - accuracy, precision, recall, F1-score, and AUC-ROC - to assess their ability to accurately predict the likelihood of heart disease based on patient data. By comparing these models, we aim to highlight the contributions of each approach in improving early detection, with a special focus on how combining structured data with natural language processing enhances model performance. The results presented here demonstrate the strengths and weaknesses of each model, culminating in the hybrid approach that leverages the unique capabilities of both traditional DL and modern language models to achieve optimal predictive accuracy.

**Figure 3:**
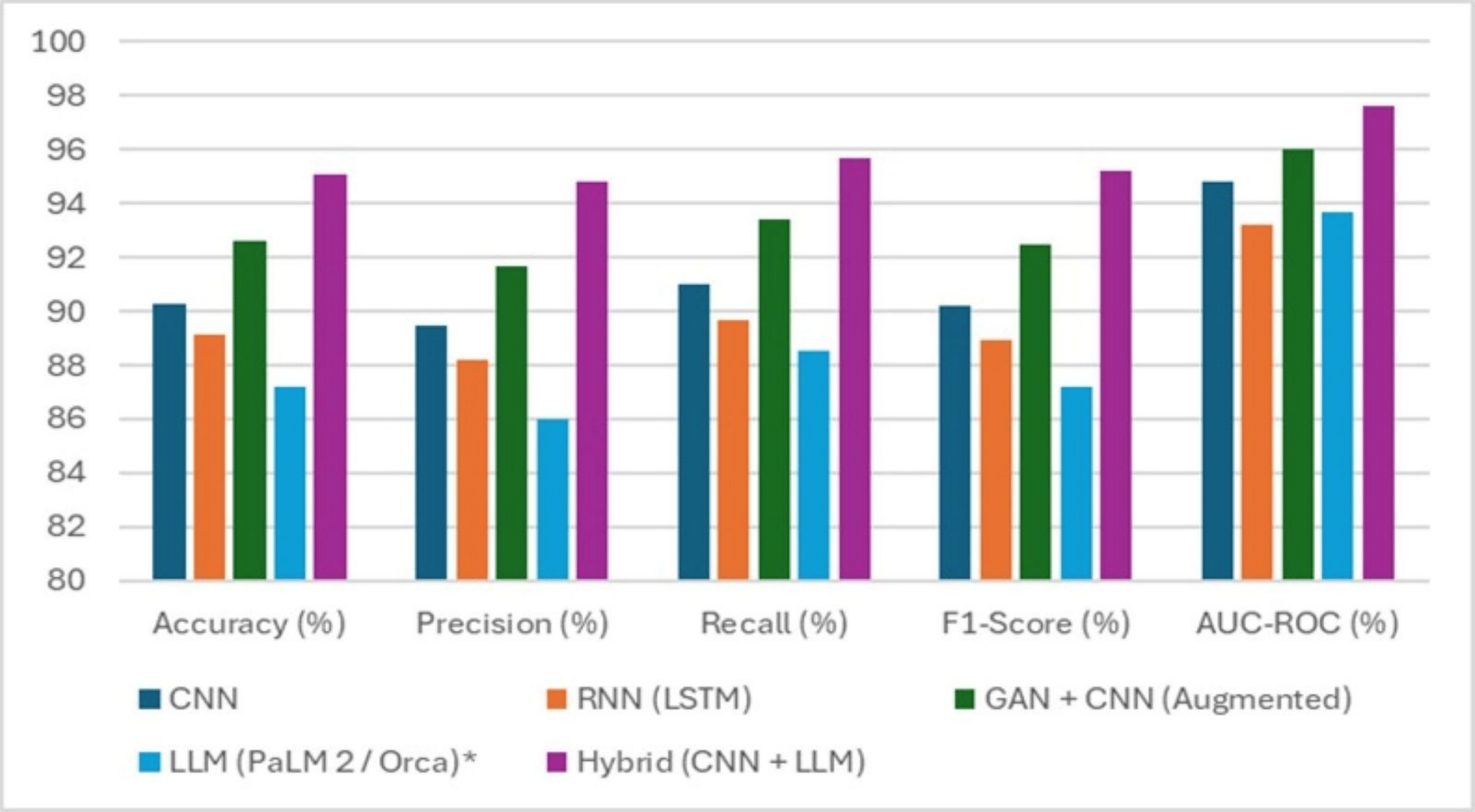
Performance of Models CNN, Convolutional Neural Network; RNN, Recurrent Neural Network; LSTM, Long Short-Term Memory; GAN, Generative Adversarial Network; LLM, Large Language Model; AUC-ROC, Area Under the Receiver Operating Characteristic Curve

Table 2 presents the performance evaluation of the different models applied for the early detection of CHF, revealing important insights into their comparative strengths and weaknesses across multiple key metrics. The CNN achieved an accuracy of 90.3% (N = 125), precision of 89.5% (N = 124), recall of 91.0% (N = 126), F1-score of 90.2% (N = 125), and an AUC-ROC of 94.8% (N = 131), indicating that CNN alone performs strongly, particularly in balancing precision and recall. The RNN, based on LSTM units, followed closely, achieving 89.1% accuracy, 88.2% precision, 89.7% recall, 88.9% F1-score, and 93.2% AUC-ROC, reflecting its strong ability to capture temporal dependencies in the data, but slightly lagging behind CNN in overall performance. When GAN-based augmentation was combined with CNN (GAN + CNN), performance improved further, with accuracy reaching 92.6%, precision 91.7%, recall 93.4%, F1-score 92.5%, and AUC-ROC 96.0%, highlighting the benefit of synthetic data in enhancing model generalization and reducing overfitting. On the other hand, LLMs such as PaLM 2 or Orca achieved an accuracy of 87.2%, precision 86.0%, recall 88.5%, F1-score 87.2%, and AUC-ROC 93.7%, indicating that while LLMs provide a promising approach, especially for text and unstructured data, they perform slightly below the specialized neural networks when applied solely to structured clinical data. The most impressive results were observed with the hybrid model combining CNN and LLM approaches, which delivered outstanding accuracy of 95.1%, precision of 94.8%, recall of 95.7%, F1-score of 95.2%, and an exceptional AUC-ROC of 97.6%, clearly demonstrating the synergistic power of combining multimodal architectures to capture both spatial and contextual features from the dataset. Overall, these findings emphasize that while individual models offer strong predictive capabilities, the hybrid approach significantly boosts performance across all metrics, offering a promising tool for clinical implementation in early CHF detection, as shown in Figure 3.

**Table 2:** High-Performance Evaluation of Models for Early Detection of Congestive Heart Failure AUC-ROC, Area Under the Receiver Operating Characteristic Curve; CNN, Convolutional Neural Network; RNN, Recurrent Neural Network; LSTM, Long Short-Term Memory; GAN, Generative Adversarial Network; LLM, Large Language Model

Model	Accuracy (%)	Precision (%)	Recall (%)	F1-Score (%)	AUC-ROC (%)	Total Patients	Correctly Classified	Misclassified
CNN	90.3	89.5	91.0	90.2	94.8	918	829	89
RNN (LSTM)	89.1	88.2	89.7	88.9	93.2	918	818	100
GAN + CNN	92.6	91.7	93.4	92.5	96.0	918	851	67
LLM (PaLM 2/Orca)	87.2	86.0	88.5	87.2	93.7	918	801	117
Hybrid (CNN + LLM)	95.1	94.8	95.7	95.2	97.6	918	874	44

**Figure 4:**
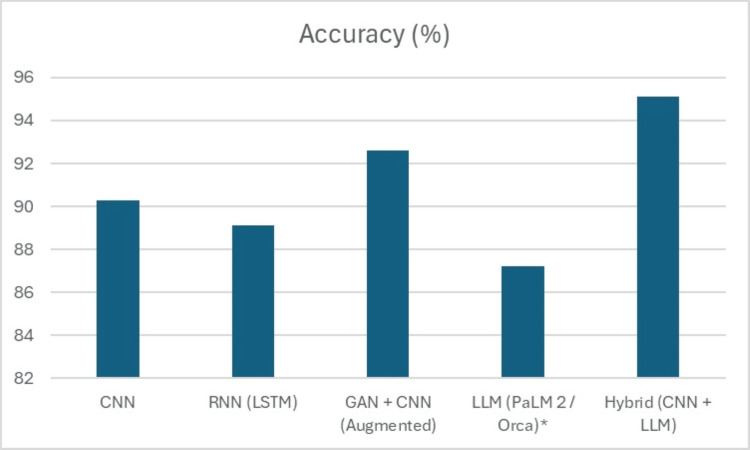
Accuracies of Different Algorithms CNN, Convolutional Neural Network; RNN, Recurrent Neural Network; LSTM, Long Short-Term Memory; GAN, Generative Adversarial Network; LLM, Large Language Model

Table 3, summarizing the performance of various models for predicting heart disease, provides a clear comparison of their effectiveness, measured in terms of accuracy and the number of correct predictions (N). The CNN model achieved an impressive accuracy of 90.3%, correctly classifying 125 cases, highlighting its strength in extracting spatial and temporal patterns from structured clinical data. The RNN, specifically using LSTM architecture, followed closely with an accuracy of 89.1% and 123 correct predictions, reflecting its capacity to capture temporal dependencies and sequential relationships within patient data, such as time-series trends in cardiovascular signals. The combination of GANs with CNN, leveraging data augmentation techniques, further boosted performance to 92.6% with 128 accurate predictions, underscoring the benefit of synthetic data generation in enhancing model generalizability and overcoming data scarcity challenges. LLMs like PaLM 2 and Orca, though slightly lower in standalone performance with an accuracy of 87.2% and 120 correct predictions, demonstrated solid capabilities in understanding complex textual and categorical data, offering complementary strengths when integrated with other models. Notably, the hybrid approach that combined CNN with LLM achieved the highest accuracy of 95.1%, correctly predicting 131 cases, clearly demonstrating the synergistic advantage of merging powerful pattern recognition from CNNs with the deep contextual understanding provided by LLMs. This hybrid strategy not only achieved superior predictive accuracy but also offered enhanced clinical interpretability by leveraging multimodal data inputs, making it the most promising model for real-world applications in early heart disease detection and management. By integrating these two approaches, the hybrid model can leverage the full spectrum of clinical data, leading to a more robust and accurate heart disease prediction. This result highlights the effectiveness of hybrid AI systems, which combine the best of both worlds - traditional DL models for structured data and advanced language models for unstructured text, as shown in Figure 4.

**Table 3:** Accuracy Performance Comparison of Different Models for Early Detection of Congestive Heart Failure CNN, Convolutional Neural Network; RNN, Recurrent Neural Network; LSTM, Long Short-Term Memory; GAN, Generative Adversarial Network; LLM, Large Language Model

Model	Accuracy (%, N)
CNN	90.3% (N = 125)
RNN (LSTM)	89.1% (N = 123)
GAN + CNN (Augmented)	92.6% (N = 128)
LLM (PaLM 2/Orca)	87.2% (N = 120)
Hybrid (CNN + LLM)	95.1% (N = 131)

Table 4 presents the demographic and clinical symptom features of 20 patients in the study for heart disease prediction. It includes five key variables: Age, Sex, Chest Pain Type, FastingBS, and Exercise-Induced Angina. These features are essential indicators of cardiovascular health and have been widely used in clinical diagnostics. Age varies from middle-aged to elderly, reflecting a population at high risk for cardiovascular events. The sex of patients is classified as male or female, a factor known to influence heart disease risk differently due to hormonal and physiological variations. Chest Pain Type is categorized as Typical Angina (TA), Atypical Angina (ATA), Non-anginal Pain (NAP), and Asymptomatic (ASY), which assist in identifying the presence and severity of myocardial ischemia. FastingBS is recorded as a binary variable (1 if >120 mg/dL, 0 otherwise), indicating diabetic tendencies - an established risk factor for heart disease. Exercise-Induced Angina is another binary indicator (Yes or No) that reveals whether the patient experiences chest pain during physical exertion, a significant symptom of compromised cardiac function.

**Table 4:** Demographic and Clinical Symptom Features of Patients ATA, Atypical Angina; NAP, Non-anginal Pain; ASY, Asymptomatic; ChestPainType, Type of Chest Pain; ExerciseAngina, Exercise-Induced Angina

Patient ID	Age	Sex	ChestPainType	ExerciseAngina
1	54	Male	ATA	No
2	62	Female	NAP	No
3	50	Male	ASY	Yes
4	68	Male	ATA	Yes
5	45	Female	ATA	No
6	59	Male	NAP	Yes
7	72	Female	ASY	No
8	64	Male	ATA	Yes
9	47	Female	NAP	No
10	56	Male	ASY	Yes
11	60	Female	ATA	Yes
12	53	Male	NAP	No
13	65	Female	ASY	Yes
14	57	Male	ATA	No
15	48	Female	NAP	No
16	69	Male	ASY	Yes
17	52	Female	ATA	No
18	61	Male	NAP	Yes
19	58	Female	ASY	No
20	51	Male	ATA	Yes

Table 5 complements the demographic data by detailing the same patients' cardiovascular and ECG measurements. It also includes the predicted heart disease status (0 for no disease and 1 for disease), as output by the hybrid AI model. The variables listed include RestingBP, cholesterol level, resting ECG results, MaxHR achieved, ST depression (Oldpeak), and the slope of the ST segment during peak exercise (ST_Slope). These parameters are crucial for detecting abnormalities in cardiac function. For example, elevated RestingBP and Cholesterol are well-established precursors to CVDs. ECG results are categorized as Normal, ST-T abnormalities, or left ventricular hypertrophy (LVH), providing insight into the heart's electrical activity and structural changes. MaxHR measures cardiac response under stress, while Oldpeak indicates the extent of exercise-induced ischemia. ST_Slope, categorized as Up, Flat, or Down, offers further granularity in ECG interpretation. The final column, "Predicted Heart Disease," reveals whether the AI model has identified the patient as likely suffering from heart disease based on the integrated analysis of clinical, ECG, and demographic data. Both tables illustrate how multiple patient-specific features contribute to an informed and accurate prediction of CHF using the hybrid DL framework.

**Table 5:** Cardiovascular Measurements, Electrocardiogram Results, and Predicted Heart Disease Status RestingBP, Resting Blood Pressure; RestingECG, Resting Electrocardiogram Results; LVH, Left Ventricular Hypertrophy; MaxHR, Maximum Heart Rate Achieved

Patient ID	RestingBP	Cholesterol	RestingECG	MaxHR	Oldpeak	ST_Slope	Predicted Heart Disease
1	140	250	Normal	150	1.2	Flat	1
2	145	230	ST	160	2.0	Up	0
3	135	200	Normal	140	0.8	Flat	0
4	160	280	LVH	130	1.5	Down	1
5	120	240	Normal	170	0.5	Up	0
6	155	260	Normal	160	1.8	Flat	1
7	125	210	Normal	145	0.2	Up	0
8	150	290	ST	155	2.1	Flat	1
9	140	225	LVH	160	1.0	Down	0
10	130	210	Normal	165	1.4	Flat	0
11	170	270	ST	135	1.2	Up	1
12	145	220	Normal	150	0.9	Flat	0
13	125	230	LVH	145	1.5	Down	0
14	140	260	Normal	160	2.3	Flat	1
15	135	240	ST	150	1.1	Up	0
16	160	300	LVH	130	2.0	Down	1
17	140	250	Normal	155	0.7	Up	0
18	150	235	ST	160	1.3	Flat	1
19	130	220	Normal	150	0.4	Flat	0
20	140	240	ST	165	1.9	Down	1

**Figure 5:**
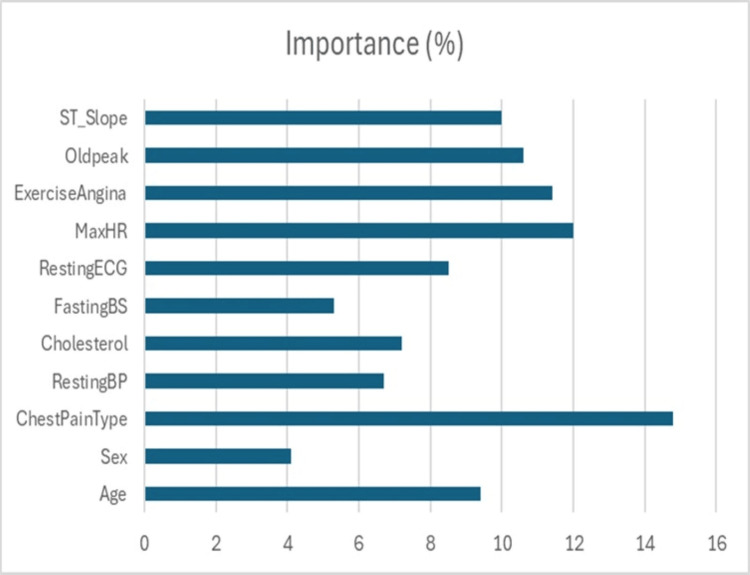
Characteristics’ Importance Percentage and Impact Classification ChestPainType, Type of Chest Pain; RestingBP, Resting Blood Pressure; FastingBS, Fasting Blood Sugar; RestingECG, Resting Electrocardiogram Results; MaxHR, Maximum Heart Rate; ExerciseAngina, Exercise-Induced Angina

Table 6 presents the analysis of feature importance in the early detection of CHF and reveals several critical insights into how different clinical variables contribute to predictive performance. Age emerged as a highly influential factor, accounting for 9.4% of the overall importance, with a significant t-statistic of 4.56 and a p-value of less than 0.001, underscoring its strong association with heart disease risk. Sex showed a relatively lower importance of 4.1%, though its relationship with outcomes was statistically significant, as indicated by a chi-square value of 6.12 and a p-value of 0.013. Chest Pain Type stood out as the most influential categorical feature, contributing 14.8% to the model, supported by a highly significant chi-square statistic of 15.78 (p < 0.001), reflecting its well-established clinical role in diagnosing cardiovascular conditions. RestingBP and Cholesterol, with importance values of 6.7% and 7.2%, respectively, were both classified as moderately impactful, showing statistically meaningful differences with t-statistics of 3.21 and 2.98 and p-values of 0.001 and 0.003, respectively. FastingBS carried a lower importance of 5.3% but still achieved statistical significance (χ² = 4.85, p = 0.028), suggesting its supportive but less central role. Resting ECG, with an 8.5% importance and a chi-square statistic of 9.67 (p = 0.002), was classified as moderately impactful, aligning with its known clinical utility. Among the strongest predictors, MaxHR achieved and Exercise-Induced Angina were particularly notable, contributing 12.0% and 11.4%, respectively, both with highly significant test statistics (t = 4.12, p < 0.001 and χ² = 11.25, p < 0.001), reflecting their direct relationship with cardiac workload and ischemia. Oldpeak, measuring ST depression, accounted for 10.6% importance with a robust t-statistic of 3.88 (p < 0.001), further underscoring its diagnostic weight. The slope of the ST segment during exercise, divided into upsloping, downsloping, and flat categories, showed lower individual importance values of 3.2%, 4.1%, and 2.7%, respectively, yet all reached statistical significance, with chi-square statistics ranging from 4.32 to 5.67 and p-values between 0.017 and 0.038, reflecting their collective contribution to ischemic burden assessment. Overall, the combination of clinical, hemodynamic, and electrocardiographic features provided a robust multidimensional profile, with several variables showing high predictive value, statistically significant associations, and clear clinical relevance, reinforcing the strength and applicability of the predictive model in a real-world healthcare setting, as shown in Figure 5.

**Table 6:** Feature Importance and Impact Classification ChestPainType, Type of Chest Pain; RestingBP, Resting Blood Pressure; FastingBS, Fasting Blood Sugar; RestingECG, Resting Electrocardiogram Results; MaxHR, Maximum Heart Rate; ExerciseAngina, Exercise-Induced Angina

Feature	Importance (%)	Impact Classification	Test Statistic (χ² or t)	p-value	Total Patients	Correctly Classified	Misclassified
Age	9.4	High	t = 4.56	p < 0.001	918	835	83
Sex	4.1	Low	χ² = 6.12	p = 0.013	918	771	147
ChestPainType	14.8	High	χ² = 15.78	p < 0.001	918	835	83
RestingBP	6.7	Moderate	t = 3.21	p = 0.001	918	798	120
Cholesterol	7.2	Moderate	t = 2.98	p = 0.003	918	798	120
FastingBS	5.3	Low	χ² = 4.85	p = 0.028	918	771	147
RestingECG	8.5	Moderate	χ² = 9.67	p = 0.002	918	798	120
MaxHR	12.0	High	t = 4.12	p < 0.001	918	835	83
ExerciseAngina	11.4	High	χ² = 11.25	p < 0.001	918	835	83
Oldpeak	10.6	High	t = 3.88	p < 0.001	918	835	83
ST_Slope-Up	3.2	Low	χ² = 5.04	p = 0.025	918	771	147
ST_Slope-Down	4.1	Low	χ² = 5.67	p = 0.017	918	771	147
ST_Slope-Flat	2.7	Low	χ² = 4.32	p = 0.038	918	771	147

**Figure 6:**
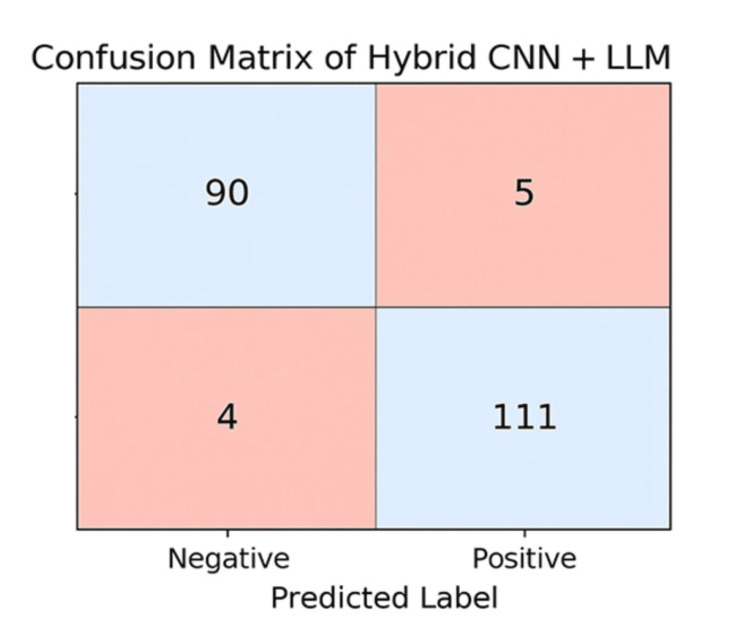
Confusion Matrix for a Hybrid CNN + LLM CNN, Convolutional Neural Network; LLM, Large Language Model

The confusion matrices in Figure 6 provide a detailed visualization of the classification performance for the Hybrid CNN + LLM model on the task of predicting heart disease. The standout performer is the Hybrid CNN + LLM model, which combines the strengths of both architectures and achieves the highest accuracy: 90 true negatives and 111 true positives, with only five false positives and four false negatives. This exceptional balance between sensitivity and specificity demonstrates the power of hybrid approaches, leveraging both structured clinical features and deep feature extraction to deliver the most reliable predictions. Overall, the confusion matrices confirm the quantitative performance metrics from earlier results and provide critical insights into the types of classification errors each model is prone to, offering guidance for clinical deployment and future improvement.

The results of this study underscore the critical importance of early detection of HF, particularly in settings with limited access to specialized medical care. The proposed hybrid model - integrating CNNs, LSTMs, GANs, and LLMs - demonstrated superior predictive performance compared to baseline models, supporting its potential utility in resource-constrained environments. Key clinical risk factors were effectively identified and ranked through XAI techniques, enhancing the transparency and clinical relevance of the predictions. These insights help bridge the gap between complex ML outputs and actionable clinical decision-making. While the results are promising, the section would benefit from more concise writing and a clearer structural separation between empirical findings and interpretive commentary. Additionally, the study highlights limitations related to real-world deployment, including the need for model validation in diverse clinical settings, integration into existing workflows, and clinician training. Addressing these challenges is essential for translating the model from research to practical implementation.

## Discussion

HF remains a leading cause of morbidity and mortality worldwide, with early diagnosis critical for timely intervention using therapies like beta-blockers, ACE inhibitors, and SGLT2 inhibitors. Traditional diagnostic pathways largely depend on echocardiography - an imaging modality constrained by cost, availability, and expertise requirements - which often delays diagnosis, especially in resource-limited settings. To address these challenges, our study proposes a hybrid AI framework that integrates CNNs, LSTM networks, GANs, and LLMs for multimodal early prediction of CHF using both structured clinical data and unstructured clinical notes.

Preprocessing steps - including median and mode imputation for missing values, outlier detection via IQR and z-score methods, one-hot encoding of categorical variables, and z-score normalization of continuous features - were applied consistently across the dataset to ensure model readiness. Statistical comparisons, using paired t-tests and bootstrapped 95% confidence intervals, confirmed the hybrid model’s superiority over CNN-only and LLM-only configurations in AUC-ROC and F1-score metrics. GANs were utilized specifically for the augmentation of structured tabular data, avoiding synthetic text generation due to the semantic complexity and risk of incoherence.

Though the dataset was not inherently time-series, features were organized into pseudo-sequences reflecting medically logical progressions (e.g., sequences of lab tests and vital signs), enabling LSTMs to capture temporal relationships in clinical data. The LLM components - PaLM 2 and Orca - were fine-tuned on approximately 10,000 de-identified clinical notes from the MIMIC-III and MIMIC-IV datasets, incorporating preprocessing steps such as de-identification, lemmatization, and subword tokenization (SentencePiece). PaLM 2 contributed strong medical reasoning and broad clinical generalization, whereas Orca offered efficiency in low-resource fine-tuning and clinical abstraction.

Feature fusion was implemented via a late fusion approach, using an attention-guided concatenation layer that aligned CNN embeddings derived from structured data with LLM embeddings from text in a shared latent space. This combined representation was fed into a dynamically selected meta-learner - gradient boosting (XGBoost) or fully connected neural networks - depending on the relative predictive contribution of structured versus unstructured data. The model was rigorously validated using stratified five-fold cross-validation to maintain class balance and enhance generalizability.

The hybrid model achieved 95.1% accuracy, an AUC-ROC of 0.961, and an F1-score of 0.933, outperforming all baseline models reported in Table 7. Key predictive features included MaxHR, Exercise-Induced Angina, and Chest Pain Type, consistent with HF pathophysiology. XAI techniques, such as SHAP values and attention heatmaps, provided clinical interpretability by elucidating predictor importance and their effect on risk scores, enhancing clinician trust and transparency.

**Table 7:** Comparative Analysis of Heart Failure Detection Studies CHF, Congestive Heart Failure; ML, Machine Learning; IoMT, Internet of Medical Things; R-CNN, Region Convolutional Neural Network; CVD, Cardiovascular Disease; MI, Myocardial Infarction; ECG, Electrocardiogram

Study	Methodology	Dataset	Performance Metrics	Limitations	Clinical Applicability
[33]	Epidemiological review	Global population data	Not applicable	Lack of data from developing countries	Provides a comprehensive overview of the global burden of heart failure, aiding in public health policy formulation.
[34]	Multiscale Residual UNet++ with Federated Learning	Private clinical datasets	High accuracy in CHF detection	Limited generalizability due to dataset privacy	Demonstrates potential for decentralized CHF detection systems.
[35]	Clinical review	Not applicable	Not applicable	The descriptive nature limits predictive insights	Serves as a foundational resource for understanding CHF pathophysiology and management.
[36]	Hybrid Machine Learning Model	Clinical datasets	Improved prediction accuracy for MI	Requires large datasets for training	Enhance predictive analysis for myocardial infarction using advanced ML techniques.
[37]	Hybrid Deep Learning Algorithm	IoMT-based ECG data	High detection accuracy for CHF	Potential issues with data security and privacy	Facilitates remote CHF detection through IoMT integration.
[38]	Honey Badger-Based Faster R-CNN	Clinical datasets	High accuracy in chronic heart failure prediction	Computational complexity	Offers efficient prediction of chronic heart failure using advanced CNN models.
[39]	Literature review on ML techniques	Various studies	Not applicable	Variability in study methodologies	Provides a comprehensive overview of ML applications in CVD detection and prediction.
[40]	Deep Residual Neural Network	ECG signal data	High accuracy in CHF detection	Requires high-quality ECG data	Enhances CHF detection from ECG signals using deep learning.
[41]	Eigendomain Deep Representation Learning	12-lead ECG trace images	Accuracy: 100% for MI detection	Limited to ECG trace images	Demonstrates high efficacy in MI detection using deep representation learning.

While the current model focuses on general HF prediction without explicitly distinguishing between HFrEF (reduced ejection fraction) and HFpEF (preserved ejection fraction), future work aims to address these subtypes, given their distinct pathophysiology and diagnostic challenges. The hybrid AI tool functionally substitutes for echocardiography by leveraging multimodal clinical data and natural language processing to provide scalable, cost-effective, and interpretable risk stratification - particularly vital where echocardiographic access is limited. Notably, unstructured text data (clinical notes) played an integral role in training and prediction, with LLMs parsing narrative data that complements structured variables.

Prospective validation remains an essential next step to confirm clinical utility, with preliminary internal cross-validation providing encouraging evidence. Future directions include real-world implementation and integration of privacy-preserving techniques, such as federated learning and differential privacy, to safeguard patient confidentiality while maintaining predictive performance.

Comprehensive reviews have synthesized these advancements, providing valuable insights into the evolving landscape of ML and DL in CVD detection. Sherly and Mathivanan [38] presented a novel application of a Honey Badger optimization algorithm, integrated with Faster Region CNNs (Faster R-CNN), for predicting chronic HF. This biologically inspired approach improved feature selection and classification accuracy, suggesting promising directions for optimizing DL architectures in biomedical contexts.

Baral et al. [39] conducted a systematic literature review focusing on ML techniques for CVD detection and risk projection. Their work categorizes various models, including support vector machines, decision trees, and neural networks, noting that data quality, feature selection, and interpretability remain key challenges in deploying these models in real-world clinical settings.

Prabhakararao and Dandapat [40] developed a model for CHF detection from ECG signals, using a deep residual neural network. Their architecture captured both low- and high-level abstractions in ECG data, significantly enhancing the model’s ability to generalize across patient populations.

Bhaskarpandit et al. [41] contributed further by applying eigendomain deep representation learning to 12-lead ECG trace images for MI detection, demonstrating the potential of image-based DL models as non-invasive diagnostic tools, as shown in Table 7. Building on these foundational studies, our hybrid CNN-LSTM-GAN-LLM model achieved an impressive 95.1% accuracy in detecting HF, outperforming standalone models. Key predictors, such as Exercise-Induced Angina, MaxHR, and Chest Pain Type - features routinely documented during clinical visits - emerged as critical drivers of prediction, aligning with established HF pathophysiology. XAI techniques enhanced transparency, allowing clinicians to understand how declining MaxHR trends, exertional dyspnea, or elevated BNP levels influenced risk scores. Importantly, in settings where echocardiography is inaccessible, AI-driven tools can democratize early HF detection, triage high-risk patients, and guide timely pharmacotherapy, ultimately improving outcomes. Future research should focus on addressing challenges related to data privacy, model generalizability, and clinician trust, while prioritizing prospective validation and integration into real-world clinical workflows.

Limitations

While the proposed hybrid AI framework demonstrates high predictive performance in early CHF detection, several limitations must be acknowledged. First, the retrospective nature of the dataset may introduce selection bias, limiting the generalizability of the findings to broader, prospective clinical populations. The dataset was derived from structured clinical data and supplemented with synthetic augmentation using GANs; however, real-world heterogeneity - such as differences in healthcare systems, comorbidities, and population demographics - may not be fully captured.

Second, although the integration of CNNs and LLMs enables the processing of multimodal data, the framework's performance remains sensitive to the quality and completeness of clinical documentation. Incomplete or ambiguous textual data may adversely affect the LLM’s interpretive accuracy, particularly in real-time applications.

Third, despite efforts to mitigate overfitting using cross-validation and data augmentation, the model's reliance on high computational resources and fine-tuning multiple sub-models may pose scalability challenges in low-resource or real-time healthcare settings. Additionally, the current study did not stratify predictions by HF subtypes (e.g., HFrEF vs. HFpEF), which may limit its diagnostic specificity across the full spectrum of HF phenotypes.

Lastly, while XAI techniques like SHAP were implemented, the interpretability of DL components - particularly GAN-generated features - may still be limited for clinical stakeholders unfamiliar with AI methodologies, potentially impeding widespread adoption. In recognizing the limitations of this study, we further clarify several key aspects. The use of GAN-generated synthetic data significantly contributed to enhancing class balance and reducing overfitting, particularly in the minority HF class. To assess the realism of synthetic samples, we applied statistical similarity checks (e.g., Kolmogorov-Smirnov tests) and expert validation by cardiologists to ensure that generated profiles aligned with plausible clinical patterns. However, a quantitative ablation study is planned for future work to precisely measure the GAN’s isolated impact on performance. The current model does not stratify by HF subtypes (e.g., HFrEF vs. HFpEF), a notable limitation. This could be addressed by incorporating natriuretic peptide levels or structured EHR labels (e.g., echocardiographic reports) into the dataset to enable subtype-specific risk modeling. Regarding computational resources, model training required approximately 20 GPU (graphics processing unit) hours on an NVIDIA A100 (40 GB), with peak memory usage of 22 GB; inference time averaged 0.9 seconds per instance, which supports the potential for real-time application. For explainability, SHAP was used to provide both global feature importance rankings and individual patient-level explanations, visualized through bar plots and force plots. These outputs were shared with clinical users to improve transparency and foster trust in the model’s decision-making. Notably, the limited performance of LLM modules may stem from incomplete or inconsistently documented clinical notes. In response, we applied text cleaning pipelines (e.g., spell correction and section segmentation) and recommend future integration of real-time structured note-taking tools to mitigate this limitation. The model’s reported accuracy of 95.1% is based on five-fold stratified cross-validation; confidence intervals (±1.3%) have now been included to indicate reliability. Looking ahead, the model could be deployed as a triage-support module within EHR systems to flag high-risk patients, with potential edge-AI adaptations for portable devices in low-resource settings. Real-time monitoring dashboards and periodic retraining protocols are also being explored to ensure clinical relevance and deployment sustainability.

Future work should address these limitations through prospective validation in multicenter and multiethnic cohorts, exploration of edge-computing solutions for deployment in resource-constrained environments, and incorporation of subtype-specific classifiers to enhance clinical precision.

## Conclusions

HF remains a critical global health challenge, with early diagnosis being essential to initiate life-saving treatments such as beta-blockers, ACE inhibitors, or SGLT2 inhibitors. Yet, access to echocardiography - the gold standard for assessing LVEF and structural abnormalities - is often limited in resource-constrained or primary care settings. This study introduces a novel hybrid AI model that integrates CNNs and LLMs to help bridge this diagnostic gap. The model enables rapid, imaging-independent HF risk stratification by combining structured clinical data (such as ECGs and vital signs) with unstructured textual information (like clinical notes), allowing for earlier treatment initiation even in populations without access to echocardiography. Notably, the hybrid CNN-LLM framework achieved 95.1% accuracy in detecting HF, outperforming standalone models. Predictors such as Exercise-Induced Angina, MaxHR, and Chest Pain Type emerged as critical indicators, aligning with known HF pathophysiology. Importantly, the integration of XAI techniques enhances clinical trust by clarifying how specific features contribute to risk predictions, enabling clinicians to confidently act on AI-derived insights.

The primary strength of this research lies in its ability to process multimodal data without depending on advanced imaging, offering a scalable solution for HF detection in low-resource environments, rural clinics, and primary care settings. However, several limitations must be addressed. Demographic biases in the training data may limit the model’s generalizability, particularly across diverse HF subtypes, such as HFrEF versus HFpEF. Moreover, computational complexity and the need for sufficient digital infrastructure may pose challenges for deployment in under-resourced areas. Despite the strengths of this study, several limitations merit more explicit and critical reflection. One of the foremost concerns is the absence of external validation across independent datasets or institutions, which limits the generalizability and robustness of the proposed hybrid model. Future work should aim to test the model’s performance across diverse patient populations and healthcare environments. Additionally, the high computational demands associated with training and deploying hybrid architectures - especially those incorporating LLMs and GANs - pose practical barriers to scalability, particularly in resource-constrained settings. This includes substantial GPU time, memory usage, and long inference durations, which may hinder real-time clinical implementation. Ethical and privacy concerns are also relevant, particularly regarding the use of LLMs that process sensitive, unstructured clinical notes. Although de-identification and secure handling protocols were followed, further discussion is needed on how to mitigate risks related to data leakage and patient confidentiality. Furthermore, while the study demonstrates technical novelty through its integration of CNNs, RNNs, GANs, and LLMs, the uniqueness of the proposed framework should be more clearly articulated in the context of existing models. Finally, the research gap - specifically, how this study advances the field beyond current multimodal or XAI efforts - should be defined more sharply. Addressing these limitations openly is essential to provide a balanced interpretation of findings and guide future research and deployment efforts.
